# Intracorporeal Versus Extracorporeal Anastomoses in Laparoscopic Right Hemicolectomy: A Single-Center Experience

**DOI:** 10.7759/cureus.44194

**Published:** 2023-08-27

**Authors:** Aizaz Khalid, Jessica Tan, Syed Mohammed Ali

**Affiliations:** 1 General Surgery, University Hospitals Sussex NHS Foundation Trust, Chichester, GBR; 2 Colorectal Surgery, University Hospitals Sussex NHS Foundation Trust, Chichester, GBR

**Keywords:** post-operative complication, laparoscopic technique, post-operative ileus, laparoscopic colorectal surgery, colorectal caner

## Abstract

Background: Right hemicolectomy is a commonly performed procedure for both benign and malignant diseases of the right colon. However, there is marked technical variation in the anastomosis technique used. In our hospital, both intracorporeal anastomosis (IA) and extracorporeal anastomosis (EA) are performed. Our study aimed to assess and compare the short-term outcomes following laparoscopic right hemicolectomies, particularly in regard to the anastomosis technique.

Methods: All consecutive adult (>18 years of age) patients who underwent elective right hemicolectomy from January 2020 to April 2023 at St Richards Hospital, Chichester, University Hospitals Sussex NHS Foundation Trust, UK, were included in our retrospective study. Data, including age at operation, body mass index, American Society of Anesthesiologists (ASA) score, pathology, type of procedure, type of anastomosis, technique of anastomosis, post-operative high-dependency unit (HDU) stay, hospital length of stay, post-operative ileus, anastomotic leak, return to theater, and in-hospital mortality, were extracted. Patients who did not get an ileocolic anastomosis, had a stoma formation, and had an open procedure or conversion to open procedure were excluded. The cases that fulfilled the criteria were shortlisted for analysis. These cases were then divided into two groups: patients who had an IA and those who had an EA.

Results: From January 2020 to April 2023, 152 patients underwent right hemicolectomy. A total of 139 patients fulfilled our eligibility criteria and were included in our final analysis. The overall mortality rate was 0.7% (1/139), the return to theater rate was 0.7% (1/139), and no anastomotic leaks were recorded. The overall ileus rate was 16.5% (23/139). The hospital length of stay was significantly longer in the EA group as compared to the IA group (p<0.004). A higher proportion (18.75%, n=21) of the patients had a recorded ileus in the EA group as compared to 7.4% (n=2) in the IA group, but this difference was not statistically significant (p=0.24).

Conclusions: We found that the patients who had IA had reduced hospital length of stay. The IA group also had clinically significant reduced rates of post-operative ileus, but this was not statistically significant. However, other short-term outcomes that were measured were similar in both groups.

## Introduction

Colon cancer is the fourth most common cancer in the United Kingdom (UK), with approximately 42,900 cases diagnosed every year [1]. Cancers of the right colon are often amenable to surgical resection if detected timely with or without adjuvant chemotherapy. The procedure carried out for resection is called right hemicolectomy, which involves the removal of the right colon, two-thirds of the transverse colon, and a variable segment of ilium.

Right hemicolectomy was first performed in 1732 by George Ronsil [2]. It was not until 1991 when the laparoscopic technique was described for this procedure [3-4]. The use of this minimally invasive technique significantly improved short-term post-operative complications in patients undergoing this major operation [5-9]. While there are many technical variations, the standard technique involved a laparoscopic-open hybrid approach in which the right colon is mobilized and the ileocolic pedicle is divided laparoscopically but delivered through an upper abdominal incision. A colectomy is then performed with an extracorporeal ileocolic anastomosis. Despite advances in technology, right hemicolectomies are associated with significant morbidity, with complication rates approaching 30% [10-11]. Post-operative ileus (1.7-13%) and anastomotic leakage (0-13.3%) are two major gastrointestinal complications leading to prolonged hospital stay and other associated morbidities [11-12]. In 2003, a technique for a complete laparoscopic right hemicolectomy was described with intracorporeal anastomosis (IA) [13]. This involves the same laparoscopic mobilization of the right colon followed by laparoscopic division of the mesentery and division of the bowel with a laparoscopic stapler. An intracorporeal side-to-side anastomosis is then created using a laparoscopic stapler, and closure of the enterotomies is performed.

In our unit, both IA and EA are performed, but all patients receive the same pre- and post- operative protocol and care. We conducted a study to assess differences in short-term outcomes between IA and EA.

## Materials and methods

Study design

This is a retrospective review of a single colorectal center in the south of England. 

Study population and data points**

All consecutive adult (>18 years of age) patients who underwent elective right hemicolectomy from January 15, 2020 to April 4, 2023 at St Richards Hospital, Chichester, University Hospitals Sussex NHS Foundation Trust, UK, were included in our study. Data, including age at operation, body mass index (BMI), American Society of Anesthesiologists (ASA) score, pathology, type of procedure, type of anastomosis, technique of anastomosis, post-operative high-dependency unit (HDU) stay, hospital length of stay, post-operative ileus, anastomotic leak, return to theater, and in-hospital mortality, were extracted using the local hospital information management system. These data were then reviewed by two authors independently for accuracy. Patients who did not have an ileocolic anastomosis, had a stoma formation, had an open procedure or conversion to open procedure were excluded. The cases that fulfilled the criteria were shortlisted for analysis. These cases were then divided into two groups: patients who had an IA and those who had an extracorporeal anastomosis (EA). 

Hospital length of stay was measured inclusive of the day of admission and discharge. Post-operative ileus was recorded if it was documented in the notes and a nasogastric tube (NG) was needed. Anastomotic leak was recorded if a diagnosis was documented in the notes or if there was a clinical suspicion of anastomotic leak documented, and this was then confirmed on radiology or intra-operatively.

Statistical analysis

Categorical variables are presented as total numbers with percentages. Continuous data are presented as mean (±standard deviation) or median (range). In all instances, the level of significance was 5% (α = 0·05). Univariate and multivariate analysis was used to check significant difference in outcomes between the two groups. IBM SPSS Statistics for Windows, version 28 (released 2021; IBM Corp., Armonk, New York, United States) was used for the statistical analysis.

## Results

From January 2020 to April 2023, 152 patients underwent right hemicolectomy. Thirteen cases were excluded: one with a planned open procedure, one with no anastomosis, and 11 with unplanned conversion from laparoscopic procedure to open procedure. A total of 139 patients fulfilled our eligibility criteria and were included in our final analysis. The demographic data of the patients are shown in Table 1. Out of the 139 patients, 80.5% (112) had an EA and 19.5% (27) had an IA. There was no significant difference in age, BMI, or ASA score between the two groups.

**Table 1:** Patient demographics and indication for right hemicolectomy. BMI, body mass index; ASA, American Society of Anesthesiologists; IBD, inflammatory bowel disease

	Extracorporeal anastomosis (n=112)	Intracorporeal anastomosis (n=27)	P-value
Age	72.10 (±12.04)	73.52 (±11.52)	0.13
BMI (kg/m^2^)	26.94 (± 5.21)	27.41 (±5.766)	0.85
ASA score			0.49
I	12	0	
II	79	20	
III	20	7	
IV	1	0	
Indication			0.48
Benign	13	4	
Polyp	7	3	
IBD	6	0	
Angiodysplasia	0	1	
Colon cancer	99	23	

Data regarding procedure-specific variables are shown in Table 2. Side-to-side anastomosis was the most common anastomotic configuration (96.7%) adopted in the EA group, followed by end-to-end anastomosis (2.6%). The most common method of EA was hand-sewn anastomosis (62.3%).

**Table 2:** Procedure-specific variables ITU, intensive treatment unit

	Extracorporeal anastomosis (n=112)	Intracorporeal anastomosis (n=27)
Anastomotic configuration		
Side to side	108 (96.4%)	27 (100%)
End to end	3 (2.6%)	
End to side	1 (0.9%)	
Anastomotic method		
Handsewn	68 (60.7%)	
Stapled	44 (39.2%)	27 (100%)
Post-operative ITU	19 (17%)	2 (7.5%)

There was one in-hospital mortality and one case of return to theater, both from the EA group. The total in-hospital mortality was 0.7% across all the patients operated. The overall rate of ileus was 16.5% (n=23) across both groups. A higher proportion (18.75%, n=21) of the patients had a recorded ileus in the EA group as compared to 7.4% (n=2) in the IA group, but this difference was not statistically significant (p=0.24). None of the patients studied had an anastomotic leak. The short-term outcomes evaluated are shown in Table 3. 

**Table 3:** Short-term outcomes after laparoscopic right hemicolectomy

	Extracorporeal anastomosis (n=112)	Intracorporeal anastomosis (n=27)	P-value
Post-operative ileus	21 (18.75%)	2 (7.4%)	0.24
Anastomotic leak	0	0	-
Return to theatre	0	0	-
In-hospital mortality	1 (0.9%)	0	-
Hospital length of stay (mean(days))	7.7	5.9	0.0037

**Figure 1:**
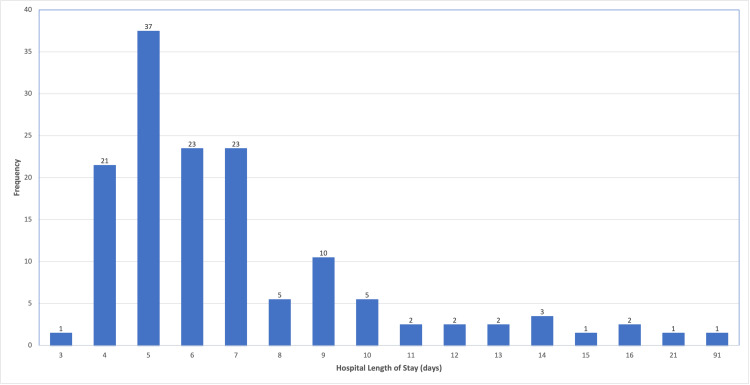
Hospital length of stay for patients undergoing laparoscopic right hemicolectomy.

A multivariate analysis with all confounding variables showed that the hospital length of stay was significantly shorter in the IA group as compared to the EA group (p=0.0037). Although a clear difference was noted between the rates of ileus in the two groups, this difference was not statistically significant. The analysis also found that the only factor that was significantly associated with post-operative ileus was post-operative HDU stay. The frequency of length of stay is depicted in Figure 1. 

## Discussion

Our study of 139 patients who underwent elective laparoscopic right hemicolectomy showed that patients who had an IA had a significantly shorter duration of stay in the hospital. No differences were observed in the rates of anastomotic leakage, return to theater, and in-hospital mortality. 

The overall rate of ileus in our study was 16.5% for both groups. A higher proportion (18.75%) of the patients in the EA group had post-operative ileus as compared to 7.4% in the IA group. Although not statistically significant, we feel that this difference is clinically very significant. Recovery of bowel function is a major determinant of discharge planning, and we hypothesize that the difference in the hospital length of stay is mediated by a reduction in the rate of ileus, particularly in subclinical/early ileus. Failure to achieve a statistically significant difference in ileus rates was likely due to our study being underpowered. 

Our results corroborate a previous work performed, and a recent meta-analysis found a similar reduction in the hospital length of stay (-0.77 days) [14]. The meta-analysis consisted of 11 retrospective studies and one prospective (non-randomized) study. All other short-term outcomes were similar in both groups.

There are only a few high-quality studies comparing IA and EA. The potential for biases is high due to the inability to adequately blind and the numbers that would be required in order to see a significant difference. Observational studies looking at both techniques in one unit are inherently biased as most surgeons will have one preferred technique. A randomized controlled trial involving 140 patients, comparing IA and EA, was performed in 2019 (Intracorporeal versus Extracorporeal Anastomosis (IEA) trial) [15]. Although they did find that IA had a shorter duration of stay, this was not statistically significant (p=0.194). In their study, however, they found a lower rate of post-operative ileus in the IA group, which was significant. The rate of anastomotic leak and re-operation was similar in both groups.

A large Italian study was conduced in 2019 (*Società Italiana di Chirurgia Endoscopica e Nuove Tecnologie* (SICE) Trial), which involved 125 surgical units [16]. Similar to our study, it found reduced hospital length of stay and reduced post-operative pain in the IA group. There were no differences in anastomotic leakage and paralytic ileus.

In 2013, the bridge between the theoretical benefits and established evidence of a total laparoscopic right hemicolectomy was first discussed by Marchesi et al. [17]. They discussed that IA has enabled the total laparoscopic approach for right hemicolectomy and hence theoretically should have replaced partial laparoscopic or EA. While there have been multiple studies demonstrating the benefits of an IA over an EA, the take-up of IA has been variable. Apprehensions to embracing the total laparoscopic approach include increased intraoperative costs, intraoperative time [15-16], and a steep learning curve. Seno et al. carried out an economic analysis of IA and EA in right hemicolectomy and found that IA leads to increased intraoperative costs [18]. They, however, concluded that the overall costs were similar. The technique also involves a learning curve and a risk of fecal spillage that makes colorectal surgeons reluctant to fully embrace IA [19]. 

IA reduces the overall length of incision required to extract the specimen. More importantly, the location of the extraction site can also be shifted from upper midline to a preferable Pfannenstiel incision, which is cosmetically better, has a reduced incidence of incisional hernia, and may reduce pulmonary compromise and post-operative pain [20]. Furthermore, an IA is thought to involve less mesenteric twisting and less subsequent bleeding and inflammation [21].

Our study presents a single-center experience of laparoscopic right hemicolectomies. Although efforts have been made to accurately present all data, there are limitations to our study. First, although our data suggest no significant difference between two groups in terms of patient selection, IA was the preferred method of operation for two of our colorectal surgeons, while others used EA. Given this, there could be an uncontrollable bias with regard to surgeon expertise, although we note that the two surgeons performing IAs comprise of the longest-serving consultant and the most newly appointed. Second, potentially relevant parameters, such as surgical site infection, operative blood loss, and operative time, were not evaluated in our study. Lastly, long-term outcomes were not assessed, which could be an important part of any future study that were to compare the two techniques.

## Conclusions

Laparoscopic right hemicolectomy with IA leads to reduced hospital length of stay as compared to EA. IA also leads to clinically significant reduced rates of post-operative ileus, although this was not statistically significant in our study. Other short-term outcomes were similar in both groups.
